# Identifying the Most Important Influencing Factors Based on Demographic Information, Nutrition, Physical Activity, Leisure Time, and Underlying Diseases in the Severity of Disease in COVID‐19 Patients Using Data Mining Techniques

**DOI:** 10.1002/hsr2.72930

**Published:** 2026-07-30

**Authors:** Noushin Mohammadifard, Nizal Sarafzadegan, Fahimeh Haghighatdoost, Jamshid Najafian, Mohammad Hossein Rouhani, Gholamreza Askari, Mohammad Sattari

**Affiliations:** ^1^ Isfahan Cardiovascular Research Center, Cardiovascular Research Institute Isfahan University of Medical Sciences Isfahan Iran; ^2^ Hypertension Research Center, Cardiovascular Research Institute Isfahan University of Medical Sciences Isfahan Iran; ^3^ Department of Community Nutrition, School of Nutrition and Food Science, Nutrition and Food Security Research Center Isfahan University of Medical Sciences Isfahan Iran; ^4^ Health Information Technology Research Center Isfahan University of Medical Sciences Isfahan Iran

## Abstract

**Background and Aims:**

The COVID‐19 pandemic has affected millions of individuals worldwide and resulted in substantial mortality. Data mining and machine learning techniques enable the analysis of comprehensive patient data, facilitating the identification of key patterns and determinants that support clinical and preventive decision‐making.

**Methods:**

This study evaluates the performance of three machine learning approaches—Deep Learning, Gradient Boosted Decision Trees (GBDT), and Support Vector Machine (SVM)—in identifying factors associated with COVID‐19 severity. A comparative analysis based on ROC curves and quantitative performance metrics demonstrates that Deep Learning and GBDT outperform SVM.

**Results:**

All models consistently identify hypertension as a major risk factor. However, differences emerge in other influential variables: GBDT ranks hypertension as the most significant factor, followed by stomach ulcers, whereas Deep Learning highlights vitamin intake as the primary determinant. The deep learning tree variant further supports the importance of vitamin consumption.

**Conclusion:**

The superior predictive performance of Deep Learning and GBDT provides a reliable benchmark and yields novel insights, including the potential roles of stomach ulcers and vitamin intake in disease severity. These findings emphasize the value of employing multiple high‐performing models and robust validation strategies for accurate identification of clinical risk factors in COVID‐19.

## 1 Introduction

The COVID‐19 pandemic is a phenomenon that has infected many people in numerous countries and has caused many deaths [1]. Despite ongoing public health efforts, the devastating impact of COVID‐19 continues to be observed worldwide. These impacts are also evident in cultural and social dimensions. The high transmission rate of this disease has disrupted normal life to such an extent that people have been prevented from attending many gatherings. Masks and social distancing have been introduced as approaches to combat this disease. These approaches have caused significant changes in businesses and have raised the topic of new technologies and innovations [2, 3].

Infection with the novel coronavirus SARS‐CoV‐2 leads to a wide range of clinical outcomes. In most cases, individuals remain asymptomatic or experience only mild symptoms, while a smaller proportion progress to severe or fatal COVID‐19 characterized by significant respiratory complications. The sudden clinical deterioration observed in critically ill patients may be associated with extensive viral involvement [4].

As of March 30, 2020, the total number of COVID‐19 cases worldwide was 693,282. Of these, 392,815 (approximately 57%) were in Europe, 142,081 (approximately 20%) in the Americas, 103,775 (approximately 15%) in the Western Pacific, 46,329 (about 7%) in the Eastern Mediterranean, 4,084 (approximately 0.5%) in South‐East Asia, and 3,486 (approximately 0.5%) in Africa. Among these cases, 33,106 deaths occurred globally, with 23,962 (about 72% of total deaths) in Europe, 3649 (about 11%) in the Western Pacific, and 5488 (around 17%) in other regions combined.

Previous studies have demonstrated that factors such as nutritional status and underlying comorbidities can significantly influence the progression and severity of COVID‐19. Dietz identified obesity and type 2 diabetes as two major risk factors associated with severe disease outcomes [5]. Butler emphasized the importance of nutrition in shaping long‐term outcomes of COVID‐19, advocating for improved access to healthy foods and greater attention to dietary habits to help mitigate long‐term complications [6]. Additionally, Paladimios reported that severe obesity, advanced age, and male sex were independently associated with increased in‐hospital mortality and poorer overall clinical outcomes [7].

Data mining and machine learning techniques enable the analysis of complex and high‐dimensional patient data, facilitating the identification of critical patterns and determinants that inform both clinical and preventive decision‐making. Methods such as multivariate logistic regression, cluster analysis, and various classification algorithms have been widely applied to derive more accurate and robust predictive factors [8, 9]. These approaches contribute to the early identification of patients at high risk of severe disease, thereby supporting more effective clinical management and pandemic control strategies.

## 2 Materials and Methods

a. Data collection method:

Models were implemented in Python 3.11 using scikit‐learn 1.4 for classical machine learning algorithms and PyTorch 2.2 for neural networks. All experiments were run on Ubuntu 22.04 with an NVIDIA RTX 4090 GPU (24 GB) and 64 GB RAM.

### 2.1 Inclusion Criteria

Patients whose condition has worsened based on vital signs and other factors related to COVID‐19, such as respiratory symptoms, and so forth.

### 2.2 Exclusion Criteria

Patients whose condition has not changed or has improved.

### 2.3 Sample Size and Sampling Method

The whole count method was used. This study will be conducted on more than 3000 people.

This secondary study will be conducted on data from the approved project implemented with the scientific code 199093 and the ethics code IR.MUI.MED.REC.1399.223 [10].

The number of patients whose data has been collected is more than 3000 people. The attributes used include demographic characteristics such as age, gender, occupation, level of education, underlying diseases such as cancer, kidney disease, cardiovascular disease, diabetes, blood pressure, as well as nutritional information such as consumption of fruit, dairy products, tea and coffee, cakes and cookies, sausages, hamburgers and pizza, as well as symptoms of COVID‐19 including cough, fever, diarrhea and vomiting, and the target variable of worsening of COVID‐19 disease. The target variable is obtained based on the follow‐up of the patient's condition and includes two states: whether the COVID‐19 disease condition has worsened or not. Before analysis, all data were anonymized by removing direct patient identifiers and transforming quasi‐identifiers to reduce re‐identification risk while preserving clinical utility. The anonymization rules were designed to preserve clinical content (symptoms, diagnoses, treatments) while minimizing re‐identification risk.

## 3 Data Preprocessing

In data preprocessing, the raw collected data are first prepared according to the problem requirements. This step usually includes removing incomplete or noisy data, normalizing or standardizing values, converting textual data into numerical form, and extracting important features to improve data quality and facilitate model training. The goal of this stage is to create clean, homogeneous, and optimized data for entering the modeling phase.

Before model training, we applied a standardized preprocessing pipeline. Continuous predictors (e.g., age, laboratory values) were z‑score normalized using statistics computed on the training data. Categorical variables were one‑hot encoded. Variables with more than 30% missing values were excluded. For the remaining predictors, missing numeric values were imputed using the median and missing categorical values with the most frequent category.

Categorical variables (e.g., admission type, ward) were encoded using one‑hot encoding. Ordinal variables (e.g., severity scores) were kept as integers. Continuous predictors were standardized as described in the preprocessing section.

We applied oversampling of the minority class using the Synthetic Minority Over‐sampling Technique (SMOTE) on the training data only. The validation and test sets were not resampled.

## 4 Modeling

### 4.1 Three Common Techniques Are Usually Applied in Modeling

Deep Learning: Deep neural networks with layered structures capable of learning complex and nonlinear features from data. These models perform particularly well in tasks such as image processing and natural language processing and are suitable for large and complex data sets [11, 12].

Gradient Boosted Decision Trees (GBDT): This method combines multiple weak decision trees that are sequentially improved. Each new tree helps reduce the errors from previous trees. GBDT is a boosting technique and is very powerful in solving prediction and classification problems with diverse and complex data [13, 14].

Support Vector Machine (SVM): This algorithm separates classes by finding the best hyperplane in the feature space. SVMs are suitable for medium to low dimensional data and for both linear and nonlinear classification problems. Using kernel functions in SVM allows handling nonlinear data [15].

In our experiments, deep learning models were configured with standard architecture, optimization, and regularization hyperparameters: number and type of layers (e.g., convolutional or fully connected), units or filters per layer, activation functions (such as ReLU or GELU), initialization scheme (e.g., He or Xavier), optimizer choice (SGD, Adam, or AdamW) together with learning rate and schedule, batch size, number of training epochs, and regularization settings including dropout rate, weight decay/L2 penalty, and early stopping criteria (patience and monitored metric). Gradient boosted tree models (e.g., XGBoost, LightGBM) were controlled by tree‑level, boosting, and regularization hyperparameters: number of trees (n_estimators), maximum tree depth (max_depth), minimum samples or child weight required to create a split, maximum number of leaves, sampling fractions for rows and columns (subsample and colsample_bytree), learning rate (shrinkage), the chosen loss function, and regularization coefficients (such as lambda and alpha) or minimum gain needed to perform a split. Support Vector Machine models were tuned via the regularization parameter *C*, the choice of kernel (linear, RBF, polynomial, sigmoid) and kernel‑specific hyperparameters including gamma (for RBF and other kernels), the polynomial degree and coef0 for polynomial or sigmoid kernels, and optional class_weight settings to compensate for imbalanced datasets.

## 5 Evaluation of Data Mining Techniques

The results from data mining techniques are evaluated using quantitative metrics such as accuracy, which indicates how well the model correctly classifies data or extracts accurate patterns. The ROC (Receiver Operating Characteristic) curve is also used as a graphical tool to assess the performance of binary classification models, showing the model's ability to correctly distinguish between positive and negative classes. This curve plots the True Positive Rate (sensitivity) against the False Positive Rate at various decision thresholds.

For evaluation, k‐fold cross validation is employed. The data are divided into k approximately equal folds. The model is then trained and tested in k iterations; in each iteration, k‐1 folds are used for training and the remaining fold for testing. This procedure ensures each fold is used once as a test set. Finally, the performance from all k iterations is averaged to provide a comprehensive and unbiased estimate of the model's quality.

This description provides a clear standard overview of data preprocessing, modeling techniques in machine learning, and model evaluation methods in the data mining process

We split the data at the patient level into training (70%), validation (15%), and test (15%) sets, ensuring that records from the same patient appeared in only one split. Hyperparameters were tuned using 5‑fold cross‑validation on the training set, and all preprocessing steps were fitted within each fold to prevent information leakage from validation or test data.

## 6 Findings

This dataset appears to include various demographic, lifestyle, and health‐related attributes. Here's a brief overview of what the data represents (Table 1).

**Table 1:** Characteristics and related values of a cardiovascular patient data set.

Attribute	Values
Job	Government, self‐employed, homemaker, retired, unemployed
Fast food consumption	Never, one to three times a month, once a week, two to three times a week, more than three times a week
Nut consumption	Never, one to three times a month, once a week, two to three times a week, more than three times a week
Protein consumption	Never, one to three times a month, once a week, two to four times a week, five to six times a week, once a day, two to three times a day, four or more times a day
Vitamin consumption	Never, one to three times a month, once a week, two to four times a week, five to six times a week, once a day, two to three times a day, four or more times a day
Fish consumption	Never, one to three times a month, once a week, two to four times a week, five to six times a week
Consumption of legumes	Never, one to three times a month, once a week, two to four times a week, five to six times a week, once a day, two to three times a day, four or more times a day
Controlled diabetes after COVID‐19	Yes, no
Physical activity level	Low, medium, high
Age	Child, adolescent, young adult, middle‐aged, elderly
Stomach ulcer severity	Low, medium, high
Left bundle branch block disorder	Present, absent
Right bundle branch block disorder	Present, absent
Annual cigarette consumption	Yes, no
Gender	Male, female
Blood pressure	Normal, high, low
Education level	Less than Bachelor, Bachelor, Master, PhD
Sitting and resting time	Low, medium, high
Infiltrative disease	Yes, no

Demographics: Job type, age groups, gender, and education level shed light on the population being studied.

Lifestyle factors: Consumption of fast food, nuts, protein, fish, smoking habits, and physical activity level indicate lifestyle and diet patterns.

Health conditions: Presence or absence of diabetes after COVID‐19, stomach ulcer severity, cardiac conduction block disorders, blood pressure levels, and infiltrative diseases indicate the health status of subjects.

Behavioral aspects: Sitting and resting time could relate to sedentary behavior.

Such a data set would be useful for studying correlations between lifestyle factors and health outcomes, especially related to cardiovascular and metabolic conditions. For example, it could be used to analyze how factors like diet, physical activity, and smoking relate to blood pressure and cardiac abnormalities, or how diabetes management post‐COVID‐19 correlates with these variables.

In terms of accuracy, the two methods of deep learning and Gradient Boosted Decision Tree performed better than the other methods. The Gradient Boosted Decision Tree method showed better performance compared to deep learning (Table 2).

**Table 2:** The accuracy of available methods.

SVM	Gradient boosting tree	Deep learning
67%	98.89%	96.62%

## 7 Results

**Figure 1:**
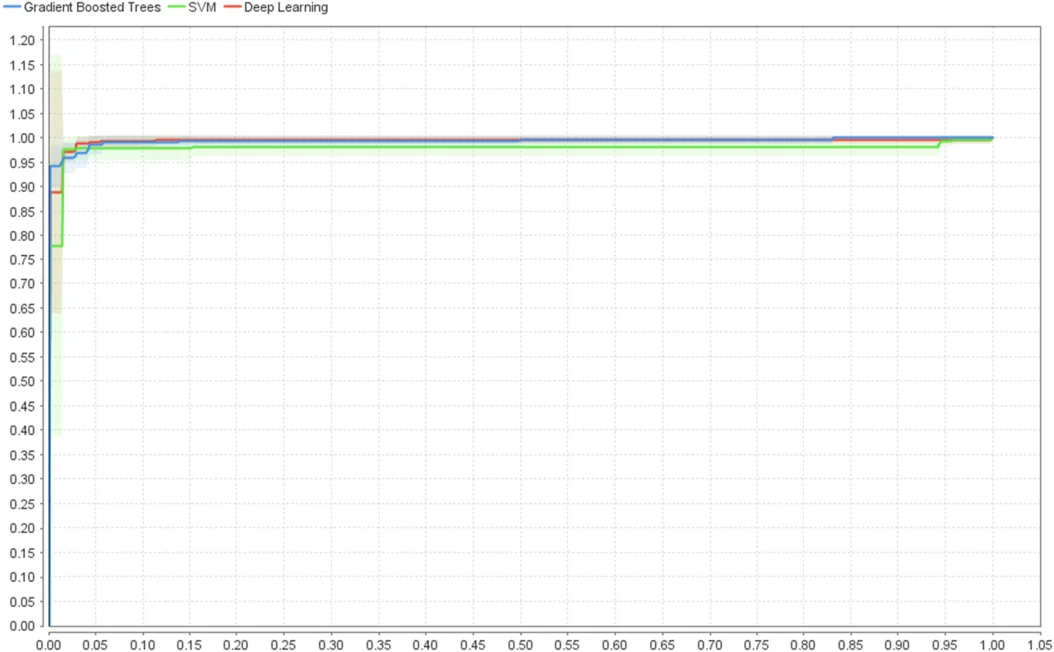
ROC curve.

According to the Figure 1, the performance of deep learning and Gradient Boosted Decision Tree (GBDT) methods is better than that of the Support Vector Machine (SVM) method. Hypertension has been identified by all three methods as one of the top three influential factors affecting COVID‐19 severity in patients (Table 3). Differences have been observed among the attributes returned by the various methods. Considering that GBDT and deep learning performed better, their results can be taken as the benchmark. From the perspective of GBDT, hypertension is the most important attribute influencing disease severity, while according to deep learning, vitamin consumption is the most influential factor affecting COVID‐19 severity. Stomach ulcers were extracted by GBDT as the second most influential factor on disease severity. This is significant because GBDT exhibited the best performance compared to other methods. Vitamin consumption was identified by deep learning as the primary factor and by the deep learning tree method as the third most impactful factor influencing COVID‐19 severity (Table 3).

**Table 3:** The most important factors affecting the severity of COVID‐19 disease.

#	Deep learning	Gradient boosting tree	SVM
1	Vitamin consumption	Blood pressure	Controlled diabetes after COVID‐19
2	Blood pressure	Stomach ulcer severity	Job
3	Controlled diabetes after COVID‐19	Vitamin consumption	Blood pressure
4	Stomach ulcer severity	Physical activity level	Fast food consumption
5	Job	Controlled diabetes after COVID‐19	Gender
6	Fast food consumption	Age	Infiltrative disease
7	Fish consumption	Fish consumption	Job
8	Sitting and Resting Time	Gender	Physical activity level
9	Nut consumption	Sitting and Resting Time	Consumption of legumes
10	Physical activity level	Fast food consumption	Right bundle branch block disorder
11	Infiltrative disease	Protein consumption	Fish consumption
12	Protein consumption	Consumption of legumes	Nut consumption
13	Amount of leisure time activity	Right bundle branch block disorder	Physical activity level
14	Right bundle branch block disorder	Annual cigarette consumption	Vitamin consumption
15	Consumption of legumes	Job	Protein consumption

## 8 Discussion

According to the findings, high blood pressure was identified by the Gradient Boosted Decision Tree (GBDT) as the most influential factor affecting the severity of COVID‐19. Additionally, controlled diabetes post‐COVID‐19 was extracted as the third most impactful factor on disease severity by the deep learning method. Research by Sabet and colleagues showed that the presence of underlying conditions such as hypertension, diabetes, and cardiovascular disorders can increase the likelihood of contracting COVID‐19 and also raise mortality rates in these patients [7]. Controlled diabetes means maintaining blood sugar levels within an acceptable range through medication, proper nutrition, and physical activity. Precise diabetes management helps reduce short‐ and long‐term complications and is particularly vital during the COVID‐19 pandemic since uncontrolled diabetic patients are more susceptible to severe forms of COVID‐19 [16]. Hypertension is a major risk factor for cardiovascular diseases and especially when combined with diabetes, increases the risk of COVID‐19 complications. Controlling blood pressure through medication, proper diet, and physical activity is very important [17, 18].

Stomach ulcers were identified by the GBDT as the second most influential factor on the severity of COVID‐19. Multiple factors such as stress, anti‐inflammatory drugs, and infections can cause stomach ulcers. These ulcers may worsen under stressful conditions of diseases like COVID‐19, and intake of vitamins and minerals can aid recovery [19].

Vitamin consumption was extracted by deep learning as the most influential factor and by the deep learning tree method as the third important factor impacting COVID‐19 severity. Proper nutrition, including intake of fruits and vegetables rich in vitamins C and A, adequate sleep, regular exercise, and avoiding tobacco, helps strengthen the immune system and prevents disease. Vitamin D plays a key role in controlling diabetes, blood pressure, and the immune system. Numerous studies have shown that vitamin D deficiency in COVID‐19 patients is associated with higher disease severity, longer hospitalization, and increased mortality. Vitamin D helps improve patient outcomes by regulating immune responses and reducing inflammation. Supplementation with vitamins C and E and minerals like magnesium also positively affects blood pressure reduction in diabetic individuals [20]. Wang represent that heightened awareness of vitamin D during the pandemic may have encouraged supplementation even among young, generally healthy adults who typically experienced only mild COVID‐19 symptoms, thereby making vitamin D‐related behavior a relevant factor to discuss in relation to lifestyle changes and bone health [21]. ŽMITEK represent that the pandemic increased vitamin D‐related health awareness among young adults, which could have influenced supplementation behaviors even in a population that was largely affected by minimal to mild COVID‐19 symptoms [22].

Physical activity was reported by the GBDT method among the top five factors influencing COVID‐19 severity. Even light regular physical activity can help improve blood glucose control, reduce blood pressure, and enhance immune function. Regular exercise also contributes to better mental health and reduces negative outcomes associated with chronic diseases and the pandemic [23]. Physical activity may also be relevant, as healthier lifestyle patterns are generally associated with better metabolic and immune function, which may contribute to more favorable COVID‐19 outcomes [24].

Together, these factors play a crucial role in maintaining overall health and enhancing quality of life. Ensuring adequate vitamin D levels, maintaining strict control of diabetes and blood pressure, managing gastric ulcers, engaging in regular physical activity, and addressing age‐related conditions all contribute to improved disease management and risk reduction—particularly in complex situations such as the COVID‐19 pandemic.

In this study, Gradient Boosted Decision Trees (GBDT) outperformed deep learning models. Although both approaches are considered highly accurate across many applications, GBDT often achieves superior performance on tabular data. In contrast, deep learning tends to perform better on other data types, such as images, text, and audio. Supporting this, prior research indicates that while deep learning excels with large‐scale and non‐tabular data, gradient boosting methods remain the preferred choice for tabular datasets and many real‐world applications.

The clinical significance of maintaining ideal blood pressure control is further highlighted by the fact that hypertension is a major cardiovascular risk factor and is linked to worse COVID‐19 results when paired with diabetes. This is particularly significant because both the pandemic setting and SARS‐CoV‐2 infection have been connected to elevated blood pressure and persistent gastrointestinal aftereffects following recovery, suggesting that blood pressure control through medication and lifestyle changes is still crucial during and after the pandemic.

## 9 Conclusion

Based on the results, the two methods of deep learning and Gradient Boosted Decision Tree (GBDT) performed better in terms of accuracy and correctness compared to other methods. In particular, GBDT identified high blood pressure as the strongest factor influencing COVID‐19 severity, while deep learning successfully extracted controlled diabetes post‐COVID‐19 as one of the key factors. This indicates that both methods have high capabilities in data analysis and identifying influential factors, playing a significant role in improving prediction of disease severity and patient treatment management. Therefore, using a combined or simultaneous approach with these two methods could enhance accuracy in recognizing impactful factors and improve therapeutic outcomes.

Overall, the findings emphasize the necessity of paying greater attention to underlying conditions like hypertension and diabetes in treatment policies and prevention strategies to reduce COVID‐19 severity. These results can contribute to better patient management strategies during the pandemic and reduce the risk of serious consequences from COVID‐19 infection.

## Author Contributions

**Noushin Mohammadifard:** conceptualization, investigation, validation, writing – review and editing. **Nizal Sarafzadegan:** conceptualization, project administration, writing – review and editing. **Fahimeh Haghighatdoost:** conceptualization, investigation, writing – review and editing, formal analysis, validation. **Jamshid Najafian:** conceptualization, formal analysis, investigation, resources, validation, Writing – review and editing. **Mohammad Hossein Rouhani:** formal analysis, validation, investigation, writing – review and editing. **Gholamreza Askari:** validation, writing – review and editing. **Mohammad Sattari:** methodology, writing – original draft, supervision.

## Ethics Statement

Research Ethics Committees of Vice‐Chancellor in Research Affairs ‐Medical University of Isfahan assign Ethical Code (IR.MUI.RESEARCH.REC.1402.134). Approval Date is 2023‐07‐03. This medical research did not involve human participants. This Medical research is corresponding to declaration of Helsinki.

## Consent

The authors have nothing to report.

## Conflicts of Interest

The authors declare no conflicts of interest.

## Transparency Statement

The Corresponding Author affirms that this manuscript is an honest, accurate, and transparent account of the study being reported; that no important aspects of the study have been omitted; and that any discrepancies from the study as planned (and, if relevant, registered) have been explained.

## Data Availability

The data that support the findings of this study are available on request from the corresponding author. The data are not publicly available due to privacy or ethical restrictions.

## References

[1] T. Singhal , “A Review of Coronavirus Disease‐2019 (COVID‐19),” Indian Journal of Pediatrics 87, no. 4 (2020): 281–286. 10.1007/s12098-020-03263-6 PMC7090728 32166607

[2] Z. Wu and J. M. McGoogan , “Characteristics of and Important Lessons From the Coronavirus Disease 2019 (COVID‐19) Outbreak in China: Summary of a Report of 72314 Cases From the Chinese Center for Disease Control and Prevention,” Journal of the American Medical Association 323, no. 13 (2020): 1239–1242. 10.1001/jama.2020.2648 32091533

[3] A. Richter , “Locked‐Down Digital Work,” International Journal of Information Management 55 (2020): 102157. 10.1016/j.ijinfomgt.2020.102157 PMC7263207 32836629

[4] J. P. Hussman , “Severe Clinical Worsening in COVID‐19 and Potential Mechanisms of Immune‐Enhanced Disease,” Frontiers in Medicine 8 (June 2021): 637642. 10.3389/fmed.2021.637642 PMC8258105 34239884

[5] W. Dietz and C. Santos‐Burgoa , “Obesity and Its Implications for COVID‐19 Mortality,” Obesity 28 (2020): 1005. 10.1002/oby.22818 32237206

[6] M. J. Butler and R. M. Barrientos , “The Impact of Nutrition on COVID‐19 Susceptibility and Long‐Term Consequences,” Brain, Behavior, and Immunity 87 (July 2020): 53–54. 10.1016/j.bbi.2020.04.040 PMC7165103 32311498

[7] L. Palaiodimos , D. G. Kokkinidis , W. Li , et al., “Study of Clinical Manifestations and Mortality Rate in Patients With COVID‐19 With Underlying Disease.” Journal of Arak University of Medical Sciences (Rahavard Danesh) (2019). 5, 743–748. 147.

[8] C. Hu , Z. Liu , Y. Jiang , et al., “Early Prediction of Mortality Risk Among Patients With Severe COVID‐19, Using Machine Learning,” International Journal of Epidemiology 49, no. 6 (2020): 1918–1929. 10.1093/ije/dyaa171 PMC7543461 32997743

[9] H. Hu , H. Du , J. Li , et al., “Early Prediction and Identification for Severe Patients During the Pandemic of COVID‐19: A Severe COVID‐19 Risk Model Constructed by Multivariate Logistic Regression Analysis,” Journal of Global Health 10, no. 2 (2020): 020510. 10.7189/jogh.10.020510 PMC7567445 33110593

[10] N. Sarrafzadegan , N. Mohammadifard , S. H. Javanmard et al., “Isfahan COVID Cohort Study: Rationale, Methodology, and Initial results,” Journal of Research in Medical Sciences 27: 65. 10.4103/jrms.jrms_552_21 PMC9639724 36353352

[11] A. Mathew , P. Amudha , and S. Sivakumari , “Deep Learning Techniques: An Overview,” In International Conference on Advanced Machine Learning Technologies and Applications 13 (February 2020): 599–608.

[12] Y. LeCun , Y. Bengio , and G. Hinton , “Deep Learning,” Nature 521, no. 7553 (2015 May 28): 436–444. 10.1038/nature14539 26017442

[13] S. Si , H. Zhang , S. S. Keerthi , D. Mahajan , I. S. Dhillon , and C. J. Hsieh , “Gradient Boosted Decision Trees for High Dimensional Sparse Output,” in International Conference on Machine Learning (July 2017), 3182–3190.

[14] Z. Zhang and C. Jung , “GBDT‐MO. Gradient‐Boosted Decision Trees for Multiple Outputs,” IEEE Transactions on Neural Networks and Learning Systems 32, no. 7 (August 2021): 3156–3167. 10.1109/TNNLS.2020.3009776 32749969

[15] M. A. Hearst , S. T. Dumais , E. Osuna , J. Platt , and B. Scholkopf , “Support Vector Machines,” IEEE Intelligent Systems and Their Applications 13, no. 4 (August 1998): 18–28.

[16] A. K. Singh , R. Gupta , A. Ghosh , and A. Misra , “Diabetes in COVID‐19: Prevalence, Pathophysiology, Prognosis and Practical Considerations,” Diabetes & Metabolic Syndrome 14, no. 4 (July 2020): 303–310. 10.1016/j.dsx.2020.04.004 PMC7195120 32298981

[17] M. Pereira , A. Dantas Damascena , L. M. Galvão Azevedo , T. de Almeida Oliveira , and J. da Mota Santana , “Vitamin D Deficiency Aggravates COVID‐19: Systematic Review and Meta‐Analysis,” Critical Reviews in Food Science and Nutrition 62, no. 5 (2022): 1308–1316. 10.1080/10408398.2020.1841090 33146028

[18] C. Argano , R. Mallaci Bocchio , M. Lo Monaco , et al., “An Overview of Systematic Reviews of the Role of Vitamin D on Inflammation in Patients With Diabetes and the Potentiality of Its Application on Diabetic Patients With COVID‐19,” International Journal of Molecular Sciences 23, no. 5 (2022): 2873. 10.3390/ijms23052873 PMC8911457 35270015

[19] K. R. Paudel , V. Patel , S. Vishwas , et al., “Nutraceuticals and COVID‐19: A Mechanistic Approach Toward Attenuating the Disease Complications,” Journal of Food Biochemistry 46, no. 12 (2022): e14445. 10.1111/jfbc.14445 PMC9874507 36239436

[20] S. Q. Abbas Rizvi , R. Ikram , S. Sarfaraz , and R. Munawwar , “Beneficial Effects of Oral Vitamin D Supplementation in Diabetes Mellitus Type II Patients—A Clinical Study in Karachi,” Pakistan Journal of Pharmaceutical Sciences 35, no. 3 (2022): 845–850. 35791486

[21] Z. Wang , A. Joshi , K. Leopold , et al., “Association of Vitamin D Deficiency With COVID‐19 Infection Severity: Systematic Review and Meta‐Analysis,” Clinical Endocrinology 96, no. 3 (2022): 281–287. 10.1111/cen.14540 PMC8444883 34160843

[22] K. Žmitek , M. Hribar , Ž. Lavriša , H. Hristov , A. Kušar , and I. Pravst , “Socio‐Demographic and Knowledge‐Related Determinants of Vitamin D Supplementation in the Context of the COVID‐19 Pandemic: Assessment of an Educational Intervention,” Frontiers in Nutrition 8 (2021): 648450. 10.3389/fnut.2021.648450 PMC8206500 34150825

[23] K. Dhuli , Z. Naureen , M. C. Medori , et al., “Physical Activity for Health,” supplement, Journal of Preventive Medicine and Hygiene 63, no. 2 Suppl 3 (2022): 150. 10.15167/2421-4248/jpmh2022.63.2S3.2756 PMC9710390 36479484

[24] K. E. Mason , G. Maudsley , P. McHale , A. Pennington , J. Day , and B. Barr , “Age‐Adjusted Associations Between Comorbidity and Outcomes of COVID‐19: A Review of the Evidence From the Early Stages of the Pandemic,” Frontiers in Public Health 9 (2021): 584182. 10.3389/fpubh.2021.584182 PMC8377370 34422736

