# Correction to “Identifying the Most Important Influencing Factors Based on Demographic Information, Nutrition, Physical Activity, Leisure Time and Underlying Diseases in the Severity of Disease in COVID‐19 Patients Using Data Mining Techniques”

**DOI:** 10.1002/hsr2.73212

**Published:** 2026-09-10

N. Mohammadifard, N. Sarafzadegan, F. Haghighatdoost, et al., “Identifying the Most Important Influencing Factors Based on Demographic Information, Nutrition, Physical Activity, Leisure Time, and Underlying Diseases in the Severity of Disease in COVID‐19 Patients Using Data Mining Techniques,” *Health Science Reports* 9 (2026): e72930, https://doi.org/10.1002/hsr2.72930.

The name of the third author was misspelled and has been updated from Fahimeh Haghighat Doost **to** Fahimeh Haghighatdoost.

The online version of this article has been corrected accordingly.

We apologize for this error.

